# Abscisic acid agonists suitable for optimizing plant water use

**DOI:** 10.3389/fpls.2022.1071710

**Published:** 2023-01-19

**Authors:** Jan Roeder, Jinghui Liu, Isabel Doch, Moritz Ruschhaupt, Alexander Christmann, Erwin Grill, Hendrik Helmke, Sabine Hohmann, Stefan Lehr, Jens Frackenpohl, Zhenyu Yang

**Affiliations:** ^1^ Department of Botany, Technical University of Munich, Freising, Germany; ^2^ Research and Development, Weed Control Research, Division Crop Science, Bayer AG, Frankfurt am Main, Germany

**Keywords:** ABA, ABA receptor, Arabidopsis, cyano cyclopropyl ABA analog, drought, transpiration, water use efficiency, wheat

## Abstract

Climate change and overexploitation of groundwater resources cause constraints on water demand for agriculture, thus threatening crop productivity. For future food security, there is an urgent need for crops of high water use efficiency combined with high crop productivity, i.e. having high water productivity. High water productivity means efficient biomass accumulation at reduced transpiration. Recent studies show that plants are able to optimize carbon uptake per water transpired with little or no trade-off in yield. The phytohormone abscisic acid (ABA) plays a pivotal role in minimizing leaf transpiration and mediating enhanced water productivity. Hence, ABA and more chemically stable ABA agonists have the potential to improve crop water productivity. Synthesis, screening, and identification of suitable ABA agonists are major efforts currently undertaken. In this study, we used yeast expressing the plant ABA signal pathway to prescreen ABA-related cyano cyclopropyl compounds (CCPs). The yeast analysis allowed testing the ABA agonists for general toxicity, efficient uptake, and specificity in regulating different ABA receptor complexes. Subsequently, promising ABA-mimics were analyzed *in vitro* for ligand-receptor interaction complemented by physiological analyses. Several CCPs activated ABA signaling in yeast and plant cells. CCP1, CCP2, and CCP5 were by an order of magnitude more efficient than ABA in minimizing transpiration of Arabidopsis plants. In a progressive drought experiment, CCP2 mediated an increase in water use efficiency superior to ABA without trade-offs in biomass accumulation.

## Introduction

Plants produce biomass using conversion of solar radiation into chemically stored energy by recruiting CO_2_ and water (Barber, 2009). The influx of atmospheric CO_2_ and the efflux of water vapor at the leaf surface share the same stomatal diffusion path (Franks et al., 2013), which inherently links CO_2_ assimilation to water vapor loss (Farquhar et al., 1989). The low abundance and the shallow gradient of CO_2_ across the stomata compared to water vapor gradients lead to exhaustive water vapor mobilization of soil-borne water into the atmosphere during CO_2_ uptake. Unlike the ubiquity of CO_2_, replenishment of freshwater resources by rainfall and aquifers varies on a large spatial and temporal scale (Rodell et al., 2018). Climate change and overexploitation of groundwater resources are expected to exacerbate the current non-sustainable water use in agriculture (Rodell et al., 2018). On a global scale, water scarcity is the dominant cause for yield losses in crops (FAO, 2021). To maintain or increase current crop productivity, there is an urgent need for water-use-efficient crops.

Water use efficiency (WUE) refers to carbon capture per unit of water consumed, however, there are several WUE levels referring to seed yield, biomass, or gas exchange (Morison et al., 2008; Blum, 2009). The ratio of net assimilation rate to stomatal conductance, the so-called intrinsic WUE (iWUE) of leaves, changes if water becomes limiting. In C3 plant species, iWUE increases by approximately two-fold during transition from well-watered conditions to dry soil (Rizza et al., 2012; Blankenagel et al., 2018). The increase is due to an appreciable reduction in transpiration while carbon capture and net photosynthesis are less negatively affected. Transpiration is reduced by diminishing stomatal aperture, which in turn, is primarily regulated by the phytohormone ABA (Raghavendra et al., 2010). ABA controls the water status of plants by adjusting the leaf gas exchange to the need for carbon availability and water availability (Yoshida et al., 2019). Under severe water deficit, ABA closes stomata and induces protective proteins and osmolytes such as Late Embryo Abundant, fibrillins, and proline together with long-term developmental adjustments (Ober and Sharp, 1994; Finkelstein and Lynch, 2000; Yang et al., 2006; Wasilewska et al., 2008). ABA is also the signal that enhances iWUE (Yang et al., 2016), therefore ABA and downstream signaling components are suitable targets for improving WUE (Blankenagel et al., 2018).

ABA is binding to Regulatory Components of ABA Receptor (RCARs)/Pyrabactin Resistance Protein1/PYR-Like proteins (PYR/PYLs) (Ma et al., 2009; Park et al., 2009). ABA stabilizes the inhibitory interaction of RCARs with ABA co-receptors that belong to type 2C protein phosphatases (PP2C). Active PP2Cs dephosphorylate and inhibit downstream-acting protein kinases of subfamily 2 related to the yeast sucrose non-fermenting kinase 1 (SnRK2) (Melcher et al., 2009; Santiago et al., 2009). In the presence of elevated ABA levels, ABA-dependent SnRK2 kinases are released from the inhibition by PP2Cs and they are activated *via* autophosphorylation (Umezawa et al., 2009; Ruschhaupt et al., 2019) and phosphorylation by RAF-like protein kinases (Lin et al., 2020; Soma et al., 2020; Takahashi et al., 2020; Lin et al., 2021). SnRK2s activate slow (S)-type anion channel (SLAC1) and inhibit inwardly-rectifying (transporting) K^+^ channel (KAT1) to induce stomatal closure (Geiger et al., 2009; Lee et al., 2009; Sato et al., 2009). In addition, these protein kinases phosphorylate and activate ABA-responsive element binding factors (AREBs/ABFs) for induction of ABA-responsive genes (Finkelstein et al., 2005; Raghavendra et al., 2010; Yoshida et al., 2010).

The *Arabidopsis thaliana* genome encodes fourteen RCAR/PYR/PYLs subgrouped into three subfamilies (Ma et al., 2009; Park et al., 2009) and nine ABA-coreceptors (Fuchs et al., 2013). Distinct ABA receptors and ABA coreceptors are able to mediate a water-use-efficient trait by subtle hyper-activation of ABA signaling under well-watered conditions (Yang et al., 2016; Yang et al., 2019). Ectopic expression of the RCAR family revealed that subfamily II members are particularly suitable for reducing transpiration without imposing a growth penalty in Arabidopsis (Yang et al., 2016; Yang et al., 2019). Similar findings are reported for wheat (*Triticum aestivum*), poplar (*Populus x canescens*), and rice (*Oryza sativa*) by overexpressing TaPYL4, PcRCAR9, and OsPYL/RCAR7, which belong to subfamily II receptors (Mega et al., 2019; Papacek et al., 2019; Bhatnagar et al., 2020). Alternatively, deficiency in PP2Cs contributes to a water-productive trait. Stacking the deficiency of the three ABA co-receptors ABI1, ABI2, and HAB1 in Arabidopsis resulted in an optimal water-saving trait with approximately 40% improved WUE comparable to RCAR6- and RCAR10-overexpressing lines (Yang et al., 2016; Yang et al., 2019).

Foliar ABA application can partially recapitulate the WUE trait achieved by genetic modification of ABA signaling. Repeated ABA administration to model and crop species resulted in moderate improvement of WUE with minor or no growth reduction both under controlled environmental and near-field conditions (Travaglia et al., 2010; Yang et al., 2019). The prospect of improving drought resistance of plants and optimizing their water use by activation of the ABA response pathway have stimulated the identification of ABA agonists by chemical synthesis and activity screening (Cao et al., 2013; Okamoto et al., 2013; Cao et al., 2017; Vaidya et al., 2017; Frackenpohl et al., 2018; Frackenpohl et al., 2019; Vaidya et al., 2019; Bojack et al., 2021). There is a long history in the synthesis of ABA agonists (Abrams and Gusta, 1989, Zaharia et al., 2005) and recently also ABA antagonistic compounds were identified (Ye et al., 2017; Vaidya et al., 2021). Synthetic ABA agonists may be related in structure to ABA such as ABA esters, aromatic ABA, and cyano cyclopropyl (CCP) ABA analogs (Zaharia et al., 2005; Benson et al., 2015; Frackenpohl et al., 2018), but also highly active ABA agonists that are derivatives of sulfonamides, phosphinamidates, phosphonamidates, and acetamides have been reported (Park et al., 2009; Cao et al., 2013; Okamoto et al., 2013; Cao et al., 2017; Vaidya et al., 2017; Frackenpohl et al., 2018; Elzinga et al., 2019; Frackenpohl et al., 2019; Vaidya et al., 2019; Frackenpohl et al., 2020; Bojack et al., 2021). Sulfonamide-type ABA agonists, such as quinabactin and its derivates, AMFs, cyanabactin, and the acetamides, 3CB and opabactin were active in reducing leaf transpiration of plants (Cao et al., 2013; Okamoto et al., 2013; Cao et al., 2017; Vaidya et al., 2017; Vaidya et al., 2019). However, most sulfonamide-type agonists are preferentially binding to dimeric ABA receptors of subfamily III, not to monomeric receptors of subfamily I and II, probably owing to the identifying screen that involved RCAR11/PYR1 and other dimeric receptors (Park et al., 2009; Okamoto et al., 2013; Vaidya et al., 2017). Among the ABA-like structures, a panel of CCPs was generated and their ABA-like activity was assessed (Frackenpohl et al., 2018). In CCPs, a cyano cyclopropyl moiety replaces the cyclohexenone ring of ABA, where the cyclopropyl group substitutes the C8’, C9’ dimethyl groups, and the cyano group mimics the carbonyl group present in the phytohormone. The combination of cyclopropyl and cyano motifs makes the CCP compounds strong bioisosteres of the natural ligand. The prototype, (2Z,4E)-6-(1-cyanocyclopropyl)-3-methyl-6-hydroxy-7-methylocta-2,4-dienoic acid (hereafter CCP1 in this study), shows an ABA-like activity both in the *in vitro* inhibition of ABI1 phosphatase activity together with RCAR11/PYR1, and as an ABA-mimic in establishing drought resistance in wheat and canola (Frackenpohl et al., 2018). These results indicate that CCP1 is a promising lead structure for identifying highly effective ABA agonists among related cyano cyclopropyl compounds.

Such ABA-agonistic chemicals synthesized for optimizing water use in agriculture are rarely characterized comprehensively in terms of their efficacy and receptor selectivity. These efforts are limited by the complexity of different ABA receptor-coreceptor combinations and by the challenges in receptor isolation for *in vitro* analysis (Tischer et al., 2017). In this study, we explored yeast expressing the plant ABA signal pathway (Ruschhaupt et al., 2019) as a simple model to prescreen various CCPs as a prelude for analyzing their potency as ABA agonists in controlling plant water relations. The yeast system provides a powerful tool for identifying ABA agonistic lead structures and their ABA receptor specificities. Several CCPs were found that are more efficient and provide longer-lasting effects than ABA in reducing transpiration and increasing WUE. These ABA agonists are promising compounds for improving crops resilience to water deficit and achieving higher water productivity.

## Materials and methods

### Chemicals and plant materials

Cyano cyclopropyl ABA analogs were provided by Bayer (Division Crop Science, Höchst, Germany). All CCPs, except CCP8, were synthesized as described (Frackenpohl et al., 2018). For the synthesis of CCP8, ethyl-(2Z)-6-(1-cyanocyclopropyl)-3-ethyl-6-hydroxy-7-methyloct-2-en-4-ynoate, copper (I) iodide (7 mg, 0.37 mmol) and bis (triphenylphosphine) palladium (II) chloride (19 mg, 0.03 mmol) were charged under argon, and toluene (3 mL) together with ethyl (2Z)-3-iodopent-2-enoate (233 mg, 0.9 mmol; column chromatography purification of a crude product as described by Frackenpohl et al., 2018) were added at room temperature. After stirring for 10 min, 1-(3-hydroxy-4-methylpent-1-yn-3-yl) cyclopropanecarbonitrile (150 mg, 0.9 mmol; synthesis as describe by Frackenpohl et al., 2018) dissolved in toluene (3 mL) and diisopropylamine (0.4 mL, 2.8 mmol) were added. The resulting reaction mixture was stirred for 3 h prior to addition of water. The aqueous phase was extracted repeatedly with dichloromethane. The combined organic phases were dried over magnesium sulphate, filtered, and concentrated under reduced pressure. Purification of the crude product by silica gel chromatography using ethyl acetate and heptane (Frackenpohl et al., 2018) recovered the ABA agonist CCP8 (167 mg, 63% of theory) as a colourless oil. ^1^H NMR (400 MHz, CDCl_3_): 6.03 (s, 1H), 4.21-4.15 (q, 2H), 2.68 (br. s, 1H, OH), 2.50-2.43 (sept, 1H), 2.30-2.26 (q, 2H), 1.47-1.41 (m, 2H), 1.33-1.30 (m, 1H), 1.30-1.28 (t, 3H), 1.25-1.21 (m, 1H), 1.16-1.11 (m, 9H). LC-MS (ret.-time, min, [M^+^/log p]) 2.84 [289.30/1.39]. HRMS-ESI: calcd for C_17_H_23_NO_3_^+^ [M+H]^+^ 289.1678, found 289.1690.

(S)-ABA was purchased from CHEMOS (www.chemos.de). Other chemicals were of the highest purity available and obtained from SigmaAldrich (www.sigmaaldrich.com), ROTH (www.carlroth.com), and J. T. Baker (http://www.avantormaterials.com) unless otherwise indicated. The Arabidopsis (*Arabidopsis thaliana*) accession Col-0 was received from the Nottingham Arabidopsis Stock Center. The elite wheat (*Triticum aestivum*) cultivar, Elixer, was provided from Bayerische Landesanstalt für Landwirtschaft, Freising. ABA and CCPs were dissolved in DMSO and diluted to designated concentrations before administration. For de-esterification, 0.2 µmol CCP was treated with 4 nkat pig liver esterase (Sigma-Aldrich, Burlington, MA, USA) for 2 h at 25°C according to supplier’s instruction.

### Plant growth conditions

Arabidopsis seeds were sown on 1/2 x MS (Murashige and Skoog) solid medium followed by stratification at 4°C for two days to promote germination as reported (Yang et al., 2016). Briefly, the germinated seeds were incubated for 7 days at 60 µmol m^-2^ s^-1^ PAR and 22°C. Single seedlings were transferred to 0.2 L pots filled with soil (Classic Profi Substrate, Einheitserde Werkverband, Sinntal-Altengronau, Germany) containing 3 mg nitrogen fertilizer per gram of dry soil. All plants were grown under an 8 h light regime with 150 µmol m^-2^ s^-1^ PAR, 22°C temperature, 50% relative humidity during the day, and 17°C and 60% at night in a plant growth cabinet (Conviron E15, Winnipeg, Canada). The short-day light regime inhibits flowering and thus allows assessment of vegetative leaf growth during the long-lasting drought experiment as well as under well-water conditions. The wheat kernels were sown directly in 0.5 L pots filled with soil as above that covered the seeds by approximately 1 cm. Wheat plants were grown as Arabidopsis with the difference of a 16 h light regime.

### Yeast growth and luciferase assay

The yeast cultivation and the life luciferase assay were performed as described (Ruschhaupt et al., 2019). Briefly, the reporter yeast expressing an ABA-dependent luciferase gene (LUC) was transformed with different combinations of ABA signaling components as indicated. Golden gate cloning (Binder et al., 2014) was used to construct pYEAST_LII_5‐6_CEN_URA vector with *TDH3* promoter and *TDH1* terminator (*tTDH1*), pYEAST_LII_1‐2_CEN_LEU vector with *GK1* promoter and *tTDH1*, and pYEAST_LII_3‐4_CEN_TRP with the *GAL1* promoter (*pGAL1*) and *tTDH1* to express RCARs, PP2Cs, and OST1 (SnRK2.6), respectively (Ruschhaupt et al., 2019). The expression of ABF2 in yeast relies on a modified pGREG503 vector, in which *pGAL1* (Jansen et al., 2005) was replaced by the *ADH1* promoter.

Colonies of freshly transformed cells were inoculated in 0.5 mL supplemented synthetic dextrose (SD) medium and incubated at 30°C in a gyratory shaker (Thermoshake-THO500, Gerhardt, Königswinter, Germany) at 200 rpm for 16-18 h. After overnight growth, the cells were harvested and washed in SD medium followed by inoculation in SD medium containing 2% galactose for induction of SnRK2 kinases under the control of the *GAL1* promoter and supplementation with ligands at indicated concentrations, to an OD600 of 0.2 in flat-bottom 96-well plates (CytoOne, Starlab, Hamburg, Germany). Yeast cultures were incubated at 30°C and 200 rpm for 16-18 h before determination of LUC activity and cell density. Light emission of yeasts was detected in a HIDEX plate luminometer (HIDEX, Turku, Finland) after injection of 100 µL freshly prepared luciferin substrate (1 mM D-luciferin, Promega) provided in 100 mM citric acid, pH 3 (Leskinen et al., 2003) for 5 s and was corrected for background. The LUC activity was normalized to the cell density (OD600) measured in 96-well flat plates (Sarstedt, Nümbrecht, Germany). LUC activity is given as the ratio of light emission to OD600 in counts per second.

### *In vitro* phosphatase assay

Expression, purification, and analysis of RCAR and PP2C proteins were performed essentially as described (Ruschhaupt et al., 2019). Briefly, the ABA receptors RCAR1, RACR8, and RCAR11 were expressed with an aminoterminal histidine tag using the pSEVA431_LII_6xHIS::RCAR1/RCAR8/RCAR11. ABA coreceptors were expressed as histidine-tagged fusions with the maltose-binding protein using the pSEVA431_LII_6xHIS::MBP::ABI2/HAB1. All constructs were transformed and expressed in *Escherichia coli* strain BL21 and purified to homogeneity according to supplier’s instruction (GE Healthcare, Chicago, USA). The receptor assay was performed with 100 nM PP2C and a two-fold molar excess of RCAR. Values are means ± SD of three replicates. In the assay, the phosphatase activity of ABI2 and HAB1 showed in the presence of RCARs and saturating ABA levels (1 mM) a residual activity of 10% and 4%, respectively. The maximum inhibition was set to 100% in the figures. The phosphatase activity was measured using 4-methylumbelliferyl-phosphate as a substrate (Meinhard and Grill, 2001). The increase in fluorescence was recorded over 25 min with excitation and emission wavelengths at 360 and 460 nm, respectively, using a Synergy 2 plate reader (Bio-Tec, Winooski, USA).

### Transient ABA response in Arabidopsis protoplasts

Preparation of Arabidopsis protoplasts, transfection of the effector DNA, and analysis of LUC activity were performed as described (Tischer et al., 2017). In essence, 10^5^ mesophyll protoplasts from leaves of the ABA deficient mutant *aba2-1* (Schwartz et al., 1997) were transfected with 4 µg DNA of the *pRD29B::LUC* construct as an ABA-responsive reporter and 3 µg of *p35S::GUS* (β-glucuronidase reporter gene) expression cassette as an internal control for normalization. In addition, 3 µg *p35S::RCAR11* or the empty vector with the same expression cassette were co-expressed with 0.1 µg *p35S::ABI2* effector constructs (Tischer et al., 2017), and the ligand-dependent induction of ABA-responsive reporter expression were assessed after incubation at 22°C. The expression of LUC and GUS was determined 18 h after transfection and ligand administration.

### Foliar administration of ABA and thermal imaging

Thermal imaging was carried out as described (Yang et al., 2016). An assessment of the efficacy of ABA and CCPs was conducted by using 35-day-old Arabidopsis and 30-day-old wheat plants. Stock solutions (10 mM) of CCPs and ABA dissolved in dimethyl sulfoxide (DMSO) were diluted to the respective ligand concentrations (10, 30, 100 µM) in water containing 0.01% (v/v) Tween 20. Aqueous Tween 20 solutions supplemented with 0.1%, 0.3%, and 1% DMSO served for mock treatments. The solutions were sprayed onto the leaf surface until the run-off of droplets. One day after application (unless otherwise indicated), thermal imaging was conducted using the InfraTec instrument (IR-TCM 384, InfraTec, Dresden, Germany). The leaf temperature of Arabidopsis was determined as the average temperature of all leaves. The leaf temperature of wheat was determined as described (Yang et al., 2019).

### Water deficit and growth

The progressive drought experiment was performed as described (Yang et al., 2016). Briefly, 18-day-old Arabidopsis plantlets grown under well-watered and short-day conditions (8 h light with 150 µmol m^-2^ s^-1^ PAR, 22°C, 50% relative humidity, and 16 h dark with 17°C, 60% relative humidity) were transferred to 0.2 L pots loaded with water-saturated soil (33.4 ± 0.4 g dry soil with 148 ± 2 g water). At the onset of the progressive drought (day 0), water was withheld, and a sealed soil surface prevented evaporation. A slowly increasing water deficit was achieved by transpirational water loss from plants. The progressive drought experiment lasted for eight weeks until mock-treated plants had consumed all the plant-usable water. At day 18, 32, and 43 the ligand solutions as indicated were administered onto the growing plants. For measuring growth and water consumption, leaf rosettes were regularly photographed, and the pots were weighed. The projected leaf area of Arabidopsis rosettes was analyzed by using Photoshop Elements software (Adobe, San Jose, USA). The consumed water was calculated as the difference in pot weight between day 0 and the designated days. The retained water was expressed as the difference between the pot weight at the end of the drought (day 57) and the weight of the loaded dry soil. Above-ground material was harvested at day 57, and the biomass was determined after drying the material for three days at 60°C to achieve constant weight. WUE was calculated as the ratio of above-ground dry biomass to consumed water. The data for the mock- and ABA-treated plants in the progressive drought experiment were already published (Yang et al., 2019).

### Statistical *a*nalysis

**Figure 1:**
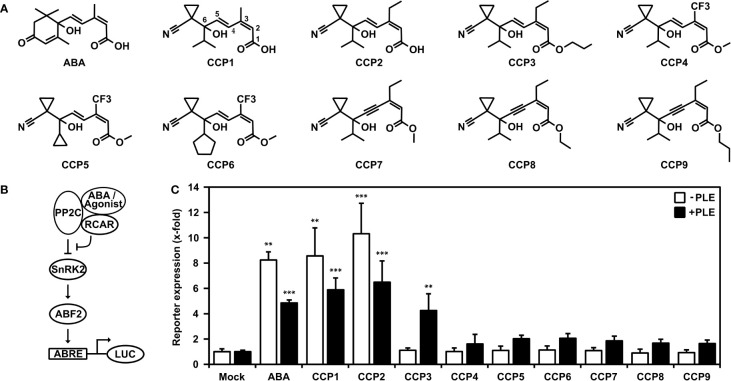
Activation of ABA signaling by ABA-related cyano cyclopropyl compounds in yeast. **(A)** Chemical structures of ABA and cyano cyclopropyl compounds CCP1 to CCP9. Carbon numbering is indicated for CCP1. **(B)** Scheme of the plant ABA signal pathway established in yeast. Binding of ABA or ABA agonists stabilizes the interaction of RCAR with the co-receptor PP2C, which inhibits the phosphatase and activates the signal pathway leading to reporter induction via the SnRK2-mediated phosphorylation of the transcription factor ABF2. The reporter gene consists of a minimal promoter with four copies of a synthetic ABA-responsive cis-element (ABRE) to drive the expression of a luciferase gene (LUC). **(C)** Reporter expression in yeast is upregulated by 30 µM ABA and CCPs. LUC activity was determined after 16 h of ligand exposure and normalized to yeast cell density. Induction of reporter response by CCPs either untreated (white bars) or treated with pig liver esterase (PLE; black bars) prior yeast exposure. The LUC activity of mock-treated cells without and with esterase pretreatment was set to 1. Mean ± SEM; n = 3 and n = 6 biological replicates, respectively. **P < 0.01, ***P < 0.001 (one-way ANOVA) compared to corresponding mock-treated yeast cells.

All data were analyzed using Microsoft Excel 365 or Origin 2020. Curve fitting in **Figure 1**, and **Supplementary Figures S2**, **S3** was achieved by using the Hill1 function. Statistical analysis was assessed by one-way ANOVA (Tukey test).

## Results

### Plant signaling pathway in yeast for analysis of ABA agonists

In this study, we used yeast combined with analyses of transient signaling in plant cells and whole plant physiology for assessment of nine structurally similar cyano cyclopropyl compounds (**Figure 1A**) including the lead structure CCP1. The other CCPs differ from CCP1 in their side chains and can be subgrouped accordingly (**Figure 1A**). CCP2 and CCP3 feature an ethyl group at C3 instead of the methyl group in CCP1 of the terpenoid side chain (**Figure 1A**, see CCP1 for C numbering), while CCP4 to CCP6 have at this position a trifluorinated methyl group (-CF3). In CCP7 to CCP9, the *trans*-ene of the side chain is replaced by an enyne group. In addition, CCP3 to CCP9 are ester derivates and in CCP5 and CCP6 the isopropyl group of CCP1 is replaced by a cyclopropyl and cyclopentyl moiety, respectively.

The reconstruction of the ABA signaling pathway in yeast offers an avenue for characterization of specific ligand-receptor complexes. The pathway consists of the ABA receptor such as RCAR11, a protein phosphatase type 2C coreceptor, e.g. ABI2, SnRK2-type protein kinase such as OST1 acting as a response mediator that phosphorylates and activates ABA-responsive transcription factors, e.g. ABF2, to drive expression of a LUC reporter gene (**Figure 1B**) in an ABA-regulated manner in yeast (Ruschhaupt et al., 2019).

Using this yeast line, 30 µM exogenous ABA or CCP1 induced the LUC reporter approximately eightfold compared to mock-treated cells (**Figure 1C**). However, only CCP2 among the other CCPs revealed an ABA-agonistic activity (**Figure 1C**). The inactive CCP3 and CCP9 are all ester derivates. The free carboxyl group of ABA is critical for binding to the ABA receptor (Santiago et al., 2009). Hence, we attempted to generate the free carboxyl group of these CCPs by incubating the compounds in the presence of an unspecific esterase, pig liver esterase (PLE) (Musidlowska-Persson and Bornscheuer, 2003). The degree of ester-bond cleavage by PLE differed among the synthetic agonists from complete de-esterification of CCP3 that convert into CCP2, to approximately 25% for CCP8 (**Supplementary Figure S1**). Examining those enzyme-treated CCPs in the yeast system showed for CCP3 a reporter activation comparable to esterase-treated CCP2 and ABA, while the other CCPs revealed only minor ABA agonistic activity (**Figure 1C**).

### CCP-dependent modulation of receptor-mediated ABA signaling in yeast

**Figure 2:**
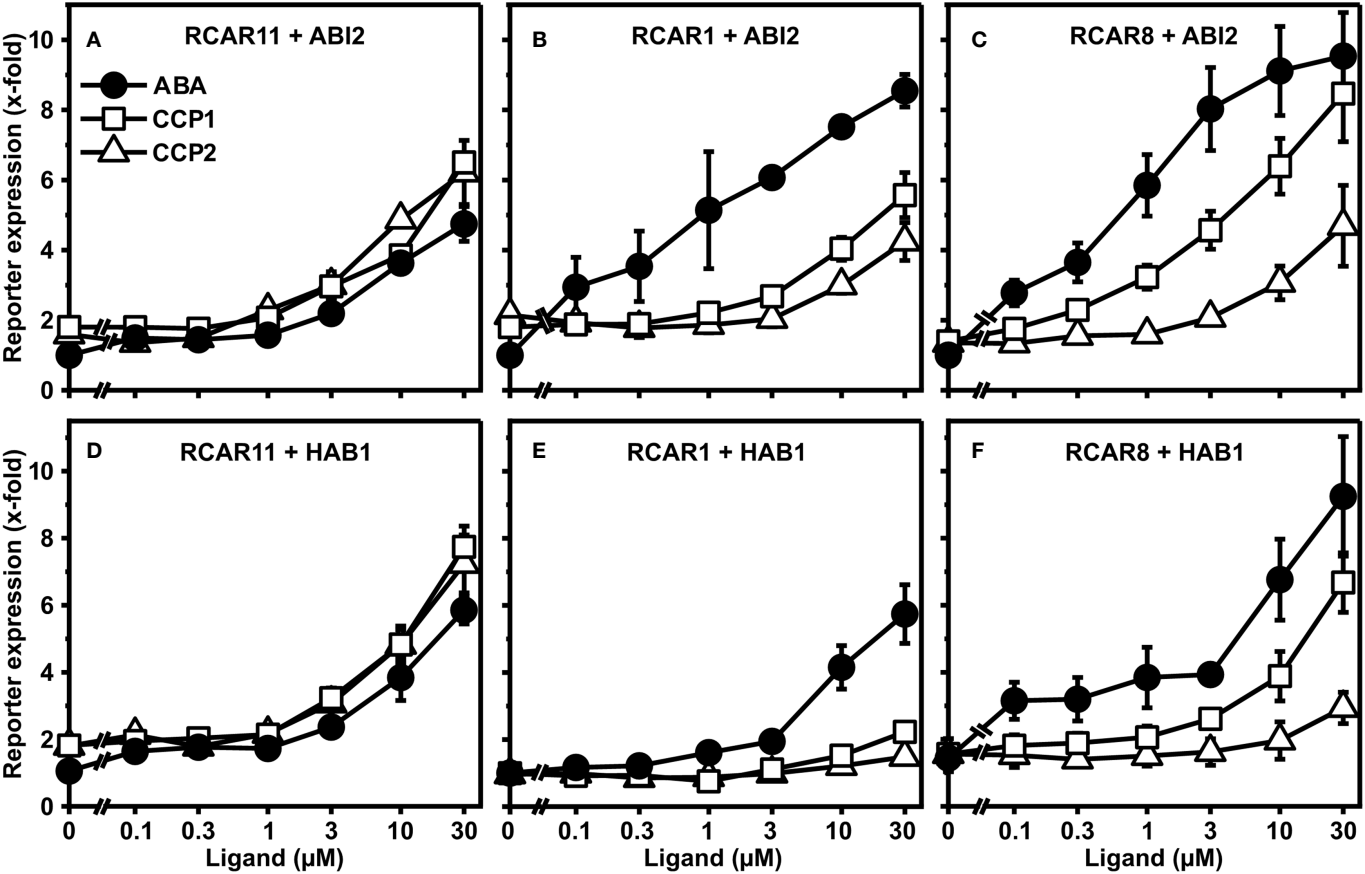
Receptor complex-dependent activation of ABA signaling by ABA, CCP1, and CCP2 in yeast. ABA- (filled circles), CCP1- (open squares) and CCP2- (open triangles) dependent regulation of ABI2 by RCAR11 **(A)**, RCAR1 **(B)**, and RCAR8 **(C)**. CCP1- and CCP2-dependent regulation of HAB1 by RCAR11 **(D)**, RCAR1 **(E)**, and RCAR8 **(F)**. n = 3 biological replicates, mean ± SEM. Statistical significance is given in **Supplementary Tables 1**, **2**.

CCP1 and CCP2 emerged as two synthetic ABA agonists with comparable activity to ABA when interacting with subgroup III receptor RCAR11. To extend these findings and to achieve a better assessment of ligand efficacy and specificity, both CCPs and ABA were analyzed for their dose-dependent activation of the ABA response pathway in yeast. In addition to RCAR11-ABI2, we also expressed in yeast the receptor RCAR1/PYL9-ABI2 and RCAR8/PYL5-ABI2 belonging to receptor subgroups I and II, respectively (**Figures 2A–C**).

Both CCPs activated the ABA response similar or even stronger than ABA *via* RCAR11-ABI2 (**Figure 2A**). In the presence of RCAR1 and RCAR8, however, the responsiveness towards both CCPs was clearly reduced compared to ABA (**Figures 2B, C**). With RCAR1, CCP1 and CCP2 required approximately 30x and >30x higher exogenous concentrations to achieve a comparable reporter expression than 1 µM ABA. In the case of RCAR8, CCP1 and CCP2 clearly differed in their capacity to regulate the ABA response with approximately half the ABA induction and no significant reporter regulation, respectively, at 1 µM exogenous ligand concentration. To achieve an induction value comparable to 1 µM ABA, about 10x and >30x higher levels of CCP1 and CCP2, respectively, were required. Exchanging ABI2 for the coreceptor HAB1 yielded similar results (**Figures 2D–F**). Both CCPs are selectively discriminated by the different receptor complexes with CCP1 being more active than CCP2 in the presence of RCAR1 and RCAR8.

### CCP1 and CCP2 are potent agonists and bind to RCAR11 with ABA-like affinity

**Figure 3:**
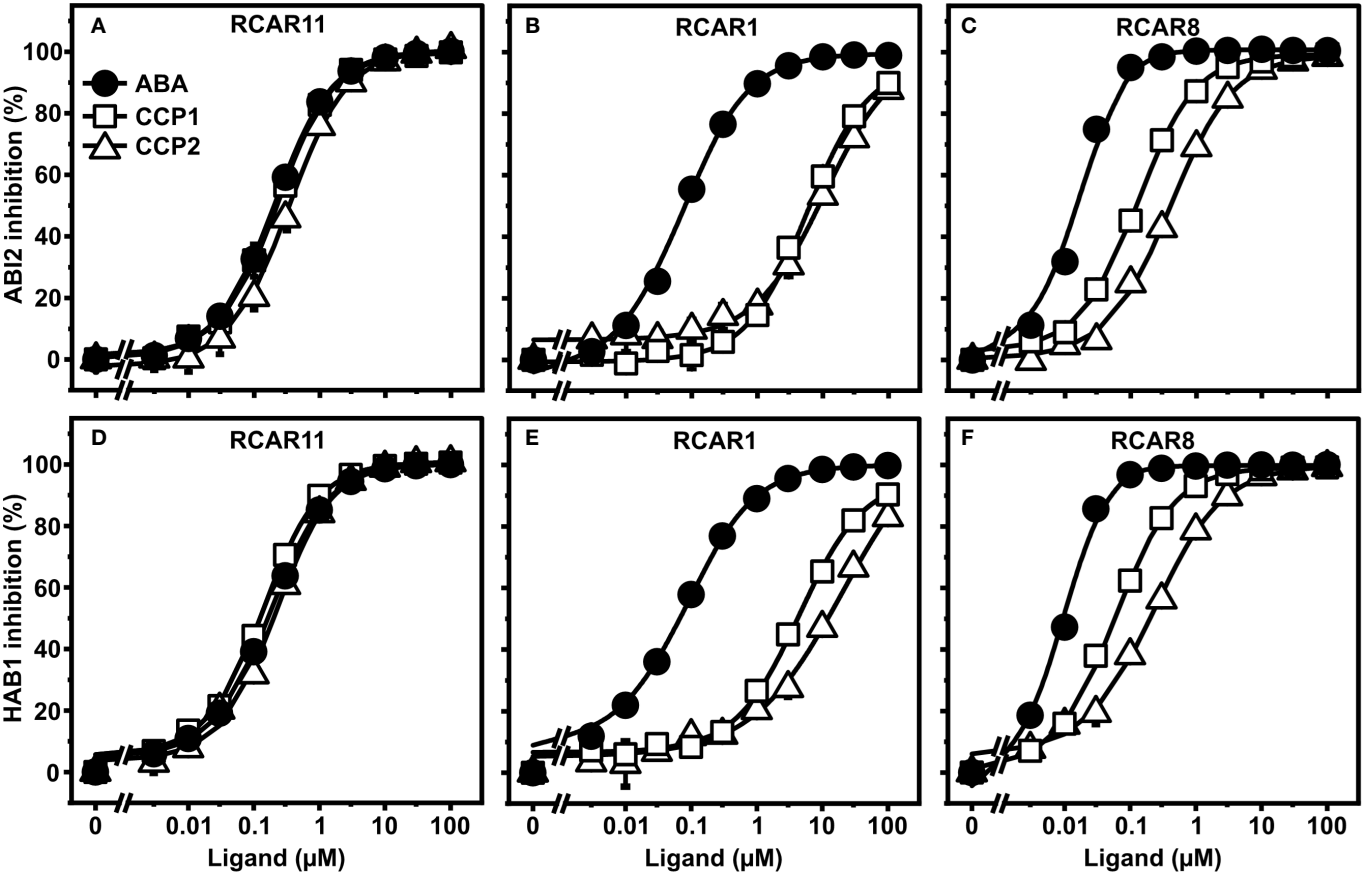
CCP1- and CCP2-mediated regulation of ABA receptor complexes. **(A–C)** ABI2 and **(D–F)** HAB1 were analyzed in combination with RCAR11 **(A, D)**, RCAR1 **(B, E)**, and RCAR8 **(C, F)** for ligand-dependent inhibition of their phosphatase activity. The in vitro analysis was performed by using 100 nM PP2C and a two-fold molar excess of RCAR at different concentrations of ABA (filled circles), CCP1 (open squares), and CCP2 (open triangles). n = 3 replicates, mean ± SD. Statistical significance is provided in **Supplementary Tables 3**–**8**.

To corroborate the ABA agonistic activity of CCPs, we next examined the compounds *in vitro* with purified receptor complexes. First, ABA, CCP1, and CCP2 were titrated to purified ABI2 together with RCAR1, RCAR8, or RCAR11 (**Figures 3A–C**). In agreement with the yeast results, CCP1 and CCP2 inhibited ABI2 in the presence of RCAR11 comparable to ABA but RCAR1 strongly discriminated against the synthetic ABA mimics (**Figures 3A, B**). For 100 nM ABI2 and 200 nM RCAR1, the half-maximal inhibitory concentration (IC50) of ABA was 100 nM whereas for CCP1 and CCP2 the values were approximately 100-fold higher (**Figure 3B**). For RCAR8-ABI2, IC50 values of CCP1 and CCP2 were around 10- and 30-fold higher than for ABA (**Figure 3C**). Assessment of the inhibition of HAB1 phosphatase activity by three RCARs and both CCPs generated similar results except a lower IC50 value for CCP2 in the presence of RCAR1 (**Figures 3D–F**). The esterified CCP3 to CCP9 compounds had no or little activity in the *in vitro* receptor assay (**Supplementary Figure S2**). Removal of the ester group by esterase treatment (**Supplementary Figure S1**) resulted in ABA-agonistic activity of CCP3 to CCP9 (**Supplementary Figure S3**). CCP3 was completely de-esterified to CCP2 and showed comparable activity as CCP2 (**Supplementary Figures S3A–C**). CCP4, CCP5, and CCP6 with a trifluorinated methyl group displayed a slightly lower activity than ABA with the RCAR11-ABI2 receptor complex, while for RCAR1 and RCAR8, IC50 values were more than 1000-fold and 100-fold higher than ABA, respectively (**Supplementary Figures S3D–F**). Enyne-containing CCP7 to CCP9 had more than 100-fold lower activity for all three receptors (**Supplementary Figures S3G–I**). Taken together, this biochemical analysis is in agreement with the yeast data revealing that CCP1, CCP2, and de-esterified CCP3 are the most active CCPs that regulate the RCAR11-ABI2 complex indistinguishable from ABA. However, CCPs are less active than ABA in other ABA receptor-coreceptor combinations.

### Regulation of ABA-responsive gene expression by CCPs in Arabidopsis protoplasts

**Figure 4:**
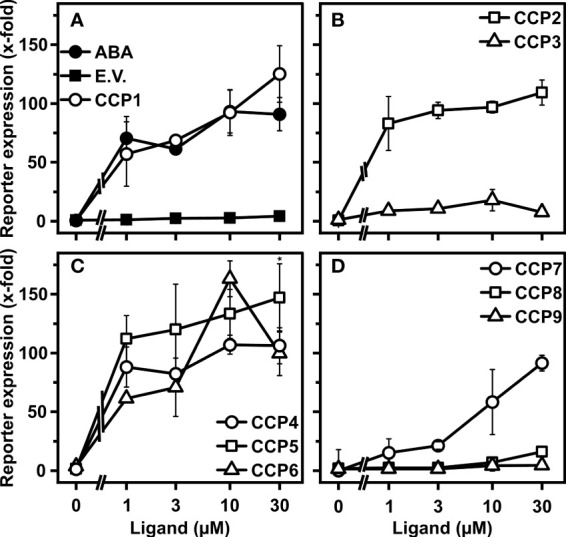
Regulation of ABA signaling in Arabidopsis cells by CCPs. **(A–D)** Arabidopsis aba2-1 protoplasts were analyzed for CCP-dependent induction of an ABA-responsive reporter. Protoplasts were transfected with DNA of several expression cassettes, including an ABA-responsive LUC reporter, a constitutively expressed GUS reporter gene for normalization, and the effector genes encoding RCAR11 and ABI2. The protoplasts were exposed to the ligands ABA and CCPs (symbols as indicated in **A–D**) for 18 h prior to analysis of reporter expression. The ABA response was expressed as fold induction relative to the value of protoplasts transfected with empty vector for the RCAR11 expression cassette (E.V.; filled squares) in the absence of ligands, which was 2.8 × 103 relative light units per relative fluorescence. Each data point represents the mean ± SD of three independent transformations plus three technical replicates per data point. **(C)** *P < 0.05 (one-way ANOVA) comparing reporter induction achieved by 30 mM CCP5 to that of 30 mM CCP4.

To what extent the CCP capacity for the ABA response activation in yeast is transferable to plants was assessed by employing plant cells and whole plant physiology. Firstly, we analyzed CCPs for stimulating ABA signaling in leaf protoplasts of the Arabidopsis mutant, *aba2-1*. ABA2 catalyzes a key step in ABA biosynthesis (Gonzalez-Guzman et al., 2002). Hence, *aba2-1* has low levels of endogenous ABA and ABA signaling is sensitively stimulated by administration of exogenous ABA (Tischer et al., 2017). Most diene-containing CCPs, CCP1 to CCP6, with the exception of CCP3 showed similar or higher activity than ABA to induce ABA signaling (**Figures 4A–C**). CCP5 was the most active compound with up to 60% higher induction rates than ABA at different ligand concentrations. Unexpectedly, the ABA-like activity of CCP3 was not observed in the protoplast assay. Since CCP3 is the ester form of CCP2 and CCP2 did show ABA-like activity in protoplasts, we anticipated that the de-esterified CCP3 should induce ABA-responsive reporter expression. Indeed, de-esterified CCP3 induced ABA-responsive reporter expression in protoplasts, similar to CCP2 and higher than ABA (**Supplementary Figure S4**). The enyne-type CCPs, CCP7 to CCP9, were not or moderately active (**Figure 4D**). The transient signaling analysis confirmed the high activity of CCP1 and CCP2, and it identified the up to now inconspicuous CCP5 as a very potent compound.

### CCPs are superior to ABA showing higher and longer-lasting efficacy in minimizing transpiration of Arabidopsis

**Figure 5:**
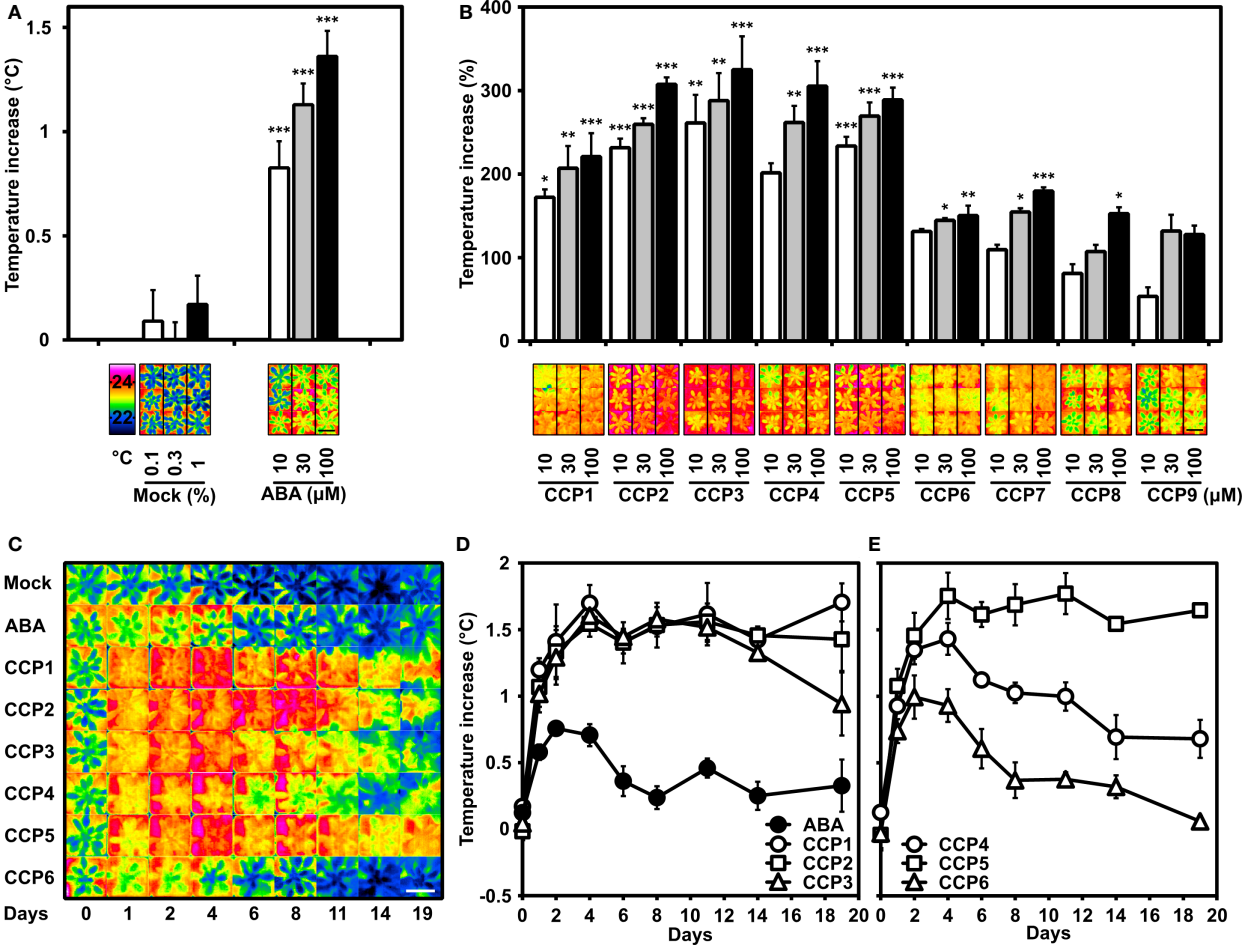
Potency of CCPs superior to ABA for regulating transpiration of Arabidopsis plants. Transpiration is indirectly assessed by determining the leaf temperatures of Arabidopsis rosettes controlled by leaf transpiration and affected by foliar ABA and ABA agonist administration. **(A)** ABA administration (10, 30, and 100 μM) was presented by spraying an aqueous solution containing the ligand and up to 1% DMSO and 0.01% (v/v) Tween 20 as solvent and surfactant, respectively. The increase in leaf temperature compared to mock-treated plants (0.1, 0.3, and 1% DMSO) and plants without treatment (set to zero) is presented. The leaf temperature difference was recorded one day after application and averaged from five independent experiments. The leaf temperature of non-treated plants was 21.9 ± 0.1°C, 22.2 ± 0.1°C, 21.4 ± 0.04°C, 21.0 ± 0.04°C, and 21.7 ± 0.1°C. Representative thermal images of mock-treated and ABA-treated plants are shown in false-colour in the lower panel together with the colour-temperature code. **(B)** The leaf temperature increase by CCP application (10, 30, and 100 μM) is expressed relative to the temperature increase achieved with 10 μM ABA (conditions as in A). The temperature increase at 10 μM ABA was 0.9 ± 0.1°C for CCP1, 0.7 ± 0.11°C for CCP2 and CCP5, 0.4 ± 0.15°C for CCP3 and CCP4, 1.1 ± 0.12°C for CCP6, and 1.0 ± 0.16°C for CCP7 to CCP9, respectively, and were set to 100% for comparison. Representative thermal images of CCPs-treated plants are shown in the lower panel. **(A, B)** 35-day-old plants were grown under short-day conditions. The white, grey, and black columns represent the data of plants either exposed to mock solvent 0.1, 0.3, 1% DMSO, or to 10, 30, 100 μM ligand solution, respectively. **(C–E)** The superior activity of selected CCPs and longer lasting efficacy than ABA is revealed by time-course experiments with a single ligand administration (100 µM; day = 0). **(C)** Representative thermal images of plants and **(D, E)** leaf temperature increases of Arabidopsis plants were recorded before treatment (day 0) and for 19 days (as indicated in C-E) after ligand administration and were compared to the mock-treated plants (22.2 ± 0.02°C; 21.4 ± 0.04°C; 22.1 ± 0.05°C; 21.9 ± 0.02°C; 22.0 ± 0.09; 21.7 ± 0.05; 21.7 ± 0.05; 21.6 ± 0.06; 20.8 ± 0.19°C). The false colour code is as shown in A). **(D,E)** Data points for plants treated with ABA and CCP1 to CCP6 are shown by symbols as indicated in the figures. **(A–E)** Single plants were cultivated in pots at short-day conditions under well-watered conditions. After treatment, the plants were randomized in the tray position. Thermal pictures of the tray with 24 pots were taken and the false-colour photograph of individual plants sorted in groups of common treatment as shown. n = 5 biological replicates per experiment, mean ± SEM. The scale bars indicate 5 cm. **(A)** ***P < 0.001 (one-way ANOVA) compared to mock treatment. **(B)** *P < 0.05, **P < 0.01, ***P < 0.001 (one-way ANOVA) compared to plants treated with 10 μM ABA.

To complement our first plant analysis of CCPs, the compounds were assayed for their efficiency in reducing leaf transpiration. Transpiration reduces the leaf temperature, which can be monitored by infrared thermal imaging (Morison et al., 2008). Our previous studies identified aqueous solutions containing 10, 30, and 100 µM ABA as suitable for changing transpiration in Arabidopsis leaves within one day (Yang et al., 2019). Hence, these concentrations of ABA and CCPs including the mock treatment were applied onto leaves of Arabidopsis grown under well-watered conditions. As reported, a dose-dependent increase in leaf temperature was observed for ABA-treated plants compared to the mock-exposed controls (**Figure 5A**). CCP administration also increased the leaf temperatures and the CCP values were compared to the temperature increase achieved by 10 µM ABA (**Figure 5B**). Most CCPs were more active than ABA. Specifically, CCP1 to CCP3 and CCP5 were the most potent. It took about 100 µM ABA to achieve a temperature increase, i.e. reduction in stomatal transpiration, similar to 10 µM CCP1 and CCP2. Interestingly, CCP3 was almost as active as the non-esterified CCP2. The CF3-containing CCPs displayed variable activity, with CCP5 bearing a cyclopropyl group at C6 being more efficacious than ABA and even exceeding the efficacy of CCP1 to CCP3, whereas CCP4 with an isopropyl group at the same position showed a moderately higher activity compared to ABA. CCP6 with a bulky cyclopentyl group at C6 displayed a somewhat lower efficacy. The enyne-type CCPs (CCP7 to CCP9) increased leaf temperatures less efficient or comparably to ABA with a clear correlation of longer alkyl ester groups impairing ABA-like activity. These results indicate several diene CCPs are by an order of magnitude more active than ABA in controlling stomatal aperture of Arabidopsis.

The higher activity of CCPs in the transpiration control possibly reflects improved uptake and higher metabolic stability of synthetic ABA mimics compared to the natural ligand. To corroborate this idea, the persistence of the minimized transpiration was examined by recording for 19 days the effect of a single application of diene CCPs (100 µM; **Figures 5C–E**). Among the analyzed chemistry, CCP1, CCP2, CCP3, and CCP5 were more active and persistent than ABA. We next chose the most active CCPs, CCP1 and CCP5, to assess their equivalency with ABA by lowering concentrations of both CCPs and comparing their effectiveness in controlling transpiration (**Supplementary Figure S5**). The ABA-inhibited transpiration reached an optimum between two and four days after administration. Subsequently, the leaf temperature dropped and reached starting values after eight days with 10 µM ABA. The 10 and 30 µM ligand treatment with CCP1 and CCP5 resulted, however, in a more persistent response for 10 days and a stronger response than with 100 µM ABA, revealing an approximately three times longer lasting effect and more than tenfold higher potency in this assay. Nineteen days after administration, plants treated with 30 µM CCP1 still had a 1.5°C higher leaf temperature clearly exceeding the maximum temperature increase triggered by 100 µM ABA (**Figure 5D** and **Supplementary Figure S5**). The results emphasize the improved potency of several diene-type CCPs in mediating long-lasting transpiration changes, more effective than ABA by at least one order of magnitude.

### Foliar administration of CCP2 and CCP3 enhance WUE in Arabidopsis

**Figure 6:**
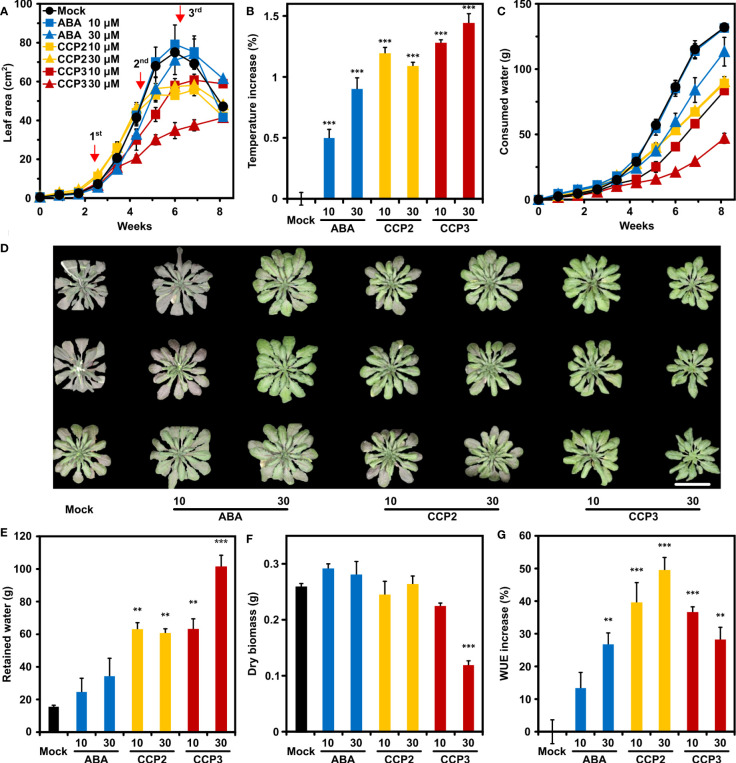
CCP2 and CCP3 are more efficient than ABA in enhancing WUE in Arabidopsis. **(A)** Kinetics of leaf growth of mock-treated plants (black), plants exposed to ABA (blue), CCP2 (yellow), or CCP3 (red) during a progressive drought experiment. The ligand solution of 10 µM (squares) and 30 µM (triangles) were administrated on day 18, 32, and 43 indicated by red arrows. The drought experiment started with cease of watering of 18-day-old plants. Growth of the plants is expressed as an increase in the projected leaf area. The decrease in leaf area was caused by leaf wilting. Statistical significance of leaf area is shown in **Supplementary Table 9**. **(B)** Leaf temperature increase of ABA- and CCPs-treated plants compared to mock-treated plants (22.5 ± 0.05°C) 24 h after the first administration. **(C)** Time course of total water consumption during the progressive drought experiment. **(D)** Wilted mock-treated plants and turgescent ligand-treated leaf rosettes at day 48. All potted plants were randomized and three representative plants for each treatment were rearranged as shown after imaging. The scale bar indicates 5 cm. At the end of the experiment at day 57, **(E)** the residual amount of soil water, **(F)** above-ground dry matter, and **(G)** the increase in WUE compared to mock-treated plants (WUE = 2.0 g L^-1^ ± 0.1 g L^-1^) was determined. The growth conditions are described in **Figure 5**, n = 4 biological replicates per treatment, mean ± SEM, **P < 0.01, ***P < 0.001 (one-way ANOVA) compared to mock-treatment.

Reduced leaf transpiration allows soil water to be saved and stored for alleviating potential incidences of water deficit. To examine the potential of CCP agonists to contribute to water saving, CCP-treated Arabidopsis was compared to mock- and ABA-challenged plants in their growth, water consumption, and WUE during a progressive drought experiment (Yang et al., 2016). CCP2 and CCP3 were chosen because they are highly efficient in increasing leaf temperature and both compounds just differ structurally in the free and ester-modified carboxyl group, respectively. The experiment started with cease of watering of eighteen-days old plants. At this stage, the total leaf area was smaller than 0.5 cm^2^, and the experiment was finished after more than eight weeks, when all available water for mock-treated plants was consumed. The mock-treated plants reached a maximum in the projected leaf area of approximately 74 cm^2^ after six weeks, followed by wilting and shrinking of the leaf rosette (**Figure 6A**). Throughout the experiment ABA and ABA agonists were three times administered by spraying 10 µM or 30 µM ligand solutions (**Figure 6A**). Growth of leaf rosettes of mock-treated and 10 µM ABA-treated plants was indistinguishable for six weeks, while 30 µM ABA, CCP2, or CCP3 treatment resulted in growth reduction (**Figure 6A**). Growth reduction of the 30 µM CCP3-challenged plants was about 60% compared to the control, suggesting a strong stomatal limitation for CO_2_ uptake. Consistently, plants sprayed with 30 µM CCP3 displayed the highest temperature increase, i.e. lowest transpiration, 24 h after the first application of ligands (**Figure 6B**). Reduced leaf growth and transpiration for ABA- or CCP-treated plants, in turn, slowed down the rate of soil water consumption compared to mock-treated plants (**Figure 6C**). Therefore, water deficit and resultant symptoms were expected to occur earlier for mock-treated plants than the ligand-treated plants. Indeed, at day 48, control plants showed first signs of wilting and leaves had a pinkish hue from stress-induced anthocyanin while the 30 µM ABA- and CCP-treated plants were turgescent and the anthocyanin induction in these plants was less pronounced (**Figure 6D**). At the end of the drought, there was still 15.2 ± 1.3 g water retained in the soil for control plants, accounting for about 10% of the initial level (**Figures 6C, E**). On the other hand, 30 µM ABA-treated plants had still about 25% of initial soil water, and approximately 75% in the case of 30 µM CCP3 treatment (**Figure 6E**). The slow growth and low water consumption of the 30 µM CCP3 cohort resulted in a high soil water content at the end of the experiment when the above-ground plant material was harvested for determination of the dry matter. Already the first week after 30 µM CCP3 administration a clear reduction in water consumption was observable while growth was little affected compared to the control (**Figures 6A, C**) supporting a higher potency of the carboxyl-alkylated compound compared to the parent CCP2. The dry-weight biomass of the ABA- and CCP2-exposed plants was similar to the control (**Figure 6F**). CCP3 treatment led to a significant reduction in biomass accumulation of 60% by 30 µM CCP3 exposure compared to the control. In terms of WUE, all ABA and ABA agonists improved the ratio of biomass per water with CCP2 doubling the benefit of ABA to almost 50% WUE increase at 30 µM solute concentration (**Figure 6G**). Taken together, these results demonstrate that foliar application of CCP2 is more effective than ABA in enhancing WUE with minor trade-offs in growth performance.

### CCPs reduce leaf transpiration of wheat

**Figure 7:**
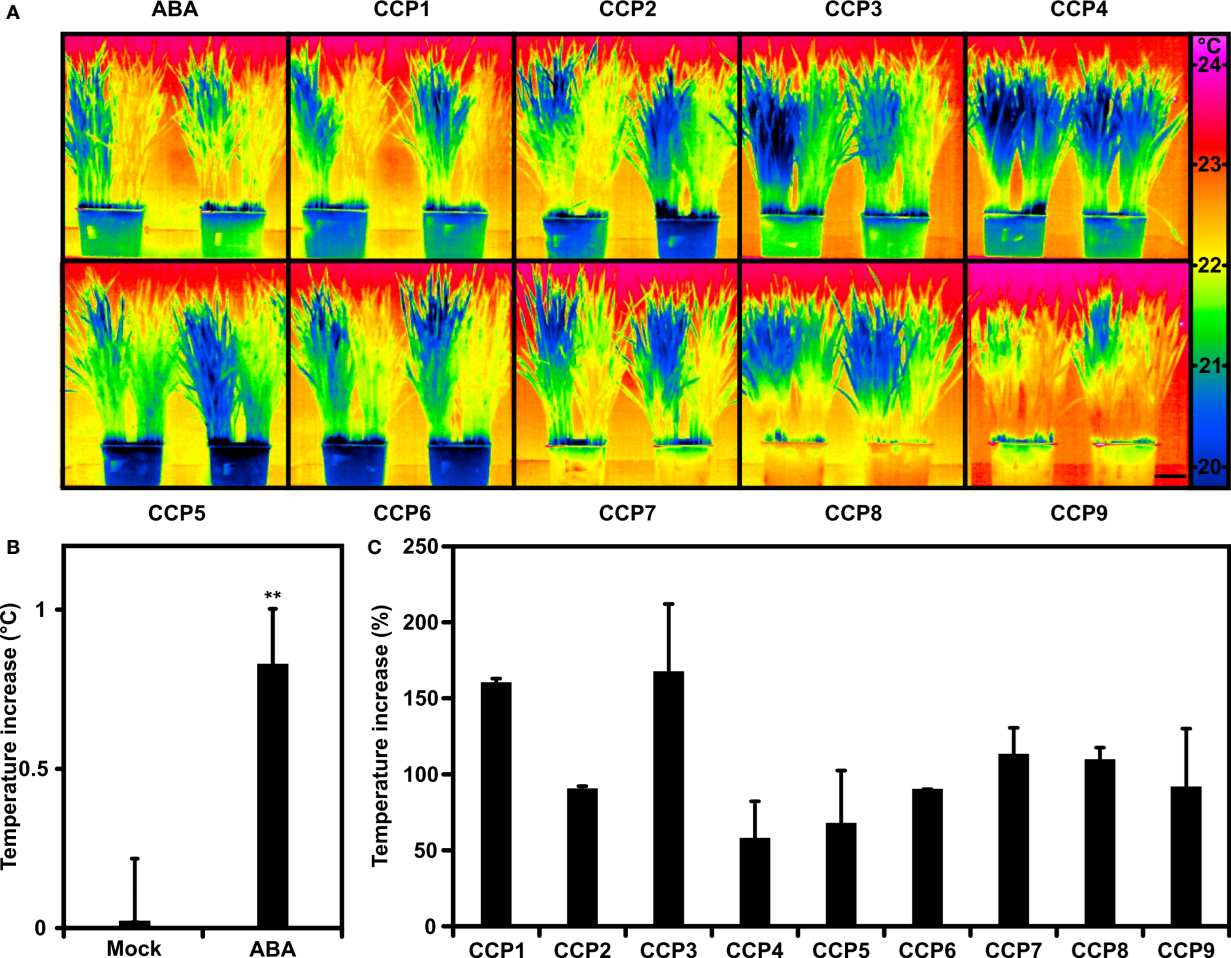
Wheat plants tuned into lowered transpiration by CCPs. **(A)** False-colour thermal images of wheat exposed to ABA and CCPs compared to mock treatment. Half of the plants grown in single pots were mock-treated with the solvent control (left half) or exposed to 100 μM ABA and ABA agonists as indicated (right half). Thermal images of 30-day-old wheat were taken 24 h after administration and two replicates are shown per treatment. **(B)** The leaf temperature increase of ABA-treated wheat in comparison to mock-treated plants. The leaf temperature difference was averaged from six independent experiments each having three biological replicates (leaf temperature of mock-treated plants, 22.1 ± 0.17°C; 20.7 ± 0.16°C; 21.9 ± 0.19°C; 21.4 ± 0.04°C; 21.7 ± 0.15°C; 21.8 ± 0.17°C). **(C)** Leaf temperature increase of CCP-treated plants relative to ABA-treated plants set to 100%. The temperature increase at 100 μM ABA was 0.6 ± 0.18°C for CCP1, 0.9 ± 0.19°C for CCP2 and CCP5, 0.6 ± 0.12°C for CCP3 and CCP4, 0.9 ± 0.03°C for CCP6, 0.9 ± 0.10°C for CCP7, and 0.9 ± 0.07°C for CCP8 to CCP9, respectively. The scale bar in **(A)** indicates 5 cm. **(B)** **P < 0.01 compared to mock-treated plants. **(C)** n = 3 biological replicates per treatment, mean ± SEM.

Arabidopsis is a dicot, whereas most of the staple crops are monocots. The question remains whether the results on CCPs gained with Arabidopsis can be transferred to a major C3 crop. Previous analysis of wheat has revealed increases of leaf temperature and WUE in response to foliar ABA administration albeit requiring higher phytohormone levels than for Arabidopsis (Yang et al., 2019). To test the CCP chemistry, wheat plants were compared in their transpiration response by recording the thermal response of wheat leaves after exposure towards 0.1 mM ABA and ABA agonists (**Figure 7**). Half the plants of a single pot were mock-treated, the other half challenged to transpiration regulators and compared to pots treated in parallel with ABA (**Figure 7A**). ABA application resulted in an approximately 0.8°C higher leaf temperature compared to the control one day after administration (**Figure 7B**). CCPs also increased the leaf temperature (**Figure 7C**). CCP1 and CCP3 had a higher activity than ABA, CCP4 and CCP5 were somewhat less active, while the other CCPs were comparable to ABA. The efficacy of CCP for rising leaf temperature differs between Arabidopsis and wheat. In both plant species, CCP1 and CCP3 were more potent than ABA, however, wheat appears to have a reduced sensitivity towards CCP5.

## Discussion

The phytohormone ABA and synthetic ABA analogs exert their physiological effects by ligand-dependent regulation of receptor-coreceptor complexes. The simultaneous expression of several RCARs and PP2C coreceptors in a single plant cell, e.g. guard cells (Yang et al., 2008), makes it difficult to assign plant responses to specific receptor complexes (Tischer et al., 2017). Synthetic ABA agonists with high selectivity for distinct receptor complexes offer the prospect of assigning ABA physiology to specific receptor complexes (Benson et al., 2015). Rebuilding ABA signaling pathways in a non-plant organism enables analyzing the specificity of the ligand for defined ABA receptor complexes and the subsequent stimulation of downstream-acting response mediators (Ruschhaupt et al., 2019).

In this study, yeast was employed with a functional ABA signaling pathway to initially characterize nine CCPs with structural similarity to ABA (**Figures 2**, **3**). The plant signaling pathway assembled in yeast consisted of the receptor complexes RCAR11-ABI2, RCAR1-ABI2, or RCAR8-ABI2 in combination with the protein kinase OST1 and the transcription factor ABF2 that activated an ABA agonist-dependent luciferase reporter gene (Ruschhaupt et al., 2019). In the yeast analysis, only CCP1 and CCP2 with a free carboxyl group were active. *In vitro* analyses with purified receptor complexes corroborated these findings (**Figures 1**–**3** and **Supplementary S2**). The carboxyl group of ABA is known to be critical for stabilizing the interaction of the ligand with the receptor in the binding pocket, as it is involved in the formation of polar contacts and water-mediated hydrogen bonds with surrounding conserved amino acid residues (Santiago et al., 2009). Esterification of the carboxyl group in ABA probably abolishes or weakens these interactions. However, charged groups frequently impair resorption, and esterification might aid in the uptake of compounds. In plants and animals, such esterified groups are readily cleaved off by unspecific esterases (Musidlowska-Persson and Bornscheuer, 2003; Cummins et al., 2007). Yeast might not contain such unspecific esterases, which could explain the general inactivity of the carboxyl-modified CCPs. Expressing unspecific esterases in yeast or simply de-esterifying CCPs before their administration is expected to overcome this limitation (Ohno and Otsuka, 1989; Frackenpohl et al., 2018). Indeed, esterase-treatment recovered variably the free acid forms of CCPs and subsequently CCP3, CCP4, CCP5, and CCP6 were similar potent as ABA to regulate the ABI2-RCAR11 receptor complex (**Supplementary Figures S1**, **S3**). For instance, esterase-treated CCP5 with approximately 60% conversion into the free acid form (**Supplementary Figure S1**) required a twofold higher concentration than ABA for half-maximum inhibition of ABI2 in complex with RCAR11 (**Supplementary Figure S3**). In the presence of RCAR1 and RCAR8, however, approximately 300- and 20-fold higher CCP5 concentrations, respectively, were necessary for inhibition comparable to the natural ligand. The results highlight the importance of structural constraints exerted by ABA agonists compared to the natural ligand contributing to the effectiveness of receptor interactions as observed for the ABA agonists pyrabactin and quinabactin that are preferentially bound to dimeric receptors (Okamoto et al., 2013; Vaidya et al., 2019). Previously, the yeast two-hybrid system has been employed to identify ligand-stabilized RCAR-PP2C interactions (Park et al., 2009; Okamoto et al., 2013; Park et al., 2015). This assay works well with subfamily III receptors such as RCAR11. The subfamily III members poorly bind to PP2Cs in the absence of the ligand in variance to subfamily I and II members (Ma et al., 2009). For instance, RCAR1 and RCAR4 expressed in yeast activated the signal pathway controlled by ABI1 in the absence of ABA (Ruschhaupt et al., 2019).

The yeast experiments and biochemical analyses were complemented by assaying plant cell ABA signaling (**Figure 4**) and whole-plant transpiration (**Figure 5**). In Arabidopsis protoplasts, the panel of CCP substances revealed ABA-agonistic activity except CCP8 and CCP9. In addition to CCP1 to CCP3, CCP5 clearly hyperactivated the response in comparison to ABA (**Figure 4C** and **Supplementary Figure S4**). CCP5 was also very active in reducing transpiration of Arabidopsis. It mediated a three times longer lasting effect than ABA, and was approximately more than ten times more potent based on the dose-dependent increase of leaf temperature similar to CCP1 (**Figure 5** and **Supplementary Figure S5**). The finding indicates an efficient CCP5 uptake and the presence of an ester-hydrolyzing activity in plants resulting in the release of a highly active ABA agonist. The superior long-term effects of several CCPs, particularly CCP1, CCP2, CCP3, and CCP5, are explainable by an improved metabolic stability compared to ABA. ABA catabolism is initiated by hydroxylation of one of the two neighboring methyl groups on the cyclohexenone moiety yielding 8’- or 9’- hydroxyl-ABA that spontaneously forms the bicyclic phaseic and neo-phaseic acid, respectively, in a reversible reaction (Kepka et al., 2011). Previously, it has been shown that the hydroxyl-ABA molecules have some ABA-like activity on isolated receptor complexes, whereas phaseic and neo-phaseic acid had no or negligible ABA activity (Kepka et al., 2011). The physiological activity of ABA is also controlled by glucosylation and de-glucosylation of the carboxyl group (Nambara and Marion-Poll, 2005). The di-methyl group of ABA is missing in CCPs and, consequently, the major route of ABA catabolism is not possible, however, glucosylation might inactivate these agonists (Frackenpohl et al., 2018).

To our knowledge, CCP analogs are the most potent synthetic ABA agonists reported up to now in reducing transpiration of Arabidopsis. Opabactin, a highly active acetamide ABA agonist, displayed an ABA-comparable efficacy in elevating Arabidopsis leaf temperature at 50 µM concentration (Vaidya et al., 2019). In our analysis, CCP1 and CCP5 were at 10 µM concentration more potent than 100 µM ABA (**Figure 5** and **Supplementary Figure S5**). Opabactin was more efficient than ABA in controlling transpiration of tomato while it had comparable activity with wheat (Vaidya et al., 2019). For wheat, CCP1 and CCP3 showed a higher efficacy than ABA but CCP5, highly potent in Arabidopsis, was less efficient. The data are consistent with species-specific differences in the bioactivity of synthetic ABA agonist. Hence, optimization of ABA chemistry requires not only structural modeling and characterization of ligand-receptor complex-interaction but testing physiological responses of target species.

Transpiration is part of the gas exchange enabling plants to take up and capture CO_2_ for photosynthesis (Farquhar and Sharkey, 1982). Hence, high yield and biomass are associated with high transpiration (Blum, 2005; Sadras et al., 2010). Indiscriminately pursuing reduction of leaf transpiration inevitably results in trade-offs in photosynthesis and plant growth. However, this issue is rarely addressed in current studies on ABA agonists (Cao et al., 2013; Okamoto et al., 2013; Cao et al., 2017; Vaidya et al., 2017; Frackenpohl et al., 2018; Vaidya et al., 2019). In this study, quantitative assessment of growth and WUE provide evidence for using CCP2 as an antitranspirant without major growth trade-offs. A single CCP2 administration restricted water consumption by approximately 30% for two weeks while growth was comparable to mock-treated plants (**Figure 6**). A second CCP2 administration inhibited growth. At the end of the drought experiment, CCP2-exposed plants yielded similar biomass levels as mock controls that had used up all plant-available soil water during drought. CCP2-exposed plants had transpired almost 70% of plant-usable soil water improving WUE by up to 50% compared to controls. Interestingly, CCP3, the methyl ester of CCP2, showed even stronger efficacy in reducing water consumption combined with a severely inhibited growth, which probably reflects insufficient CO_2_ influx caused by improved uptake of the ester compared to CCP2.

The balance between transpiration restriction and growth trade-off is delicate. If CO_2_ is the growth limiting factor, lowering of CO_2_ availability at the site of photosynthetic carboxylation results in lowered photosynthesis rates (Condon, 2020). This is a trivial conclusion. At the whole-plant physiological level, which includes adaptive responses to water deficit (still hardly understood), however, plants are able to compensate, at least partially, for restricted water availability and consequently lowered stomatal conductance (Yang et al., 2016; Yang et al., 2019; Baca Cabrera et al., 2020). Depending on water pressure deficit, up to 40% higher WUE was observed by subtle hyperactivation of ABA signaling in Arabidopsis and no-growth trade-off for Arabidopsis growing at 22°C and 50% relative humidity (Yang et al., 2016). Long-term plant growth analyses provide a sensitive readout for minor changes in the energetics of biomass accumulation. For instance, a two percent reduction in daily biomass accumulation, hardly detectable by instant measurements, results in almost 70% decreased biomass after eight weeks of growth. While application of 10 µM ABA did not affect growth relative to the mock-treated plants over more than three weeks (**Figure 6**), 30 µM ABA administration resulted in a 20% reduced projected leaf area within 19 days accounting for a 1% daily growth reduction. The two different ABA treatments increased WUE by 14% and 26% allowing plants to produce more biomass per water. Similar results were obtained for wheat (Yang et al., 2019). The findings are a promising starting point for achieving more water productivity in the field. ABA agonists like CCPs with increased physiological stability, improved resorption, and higher receptor complex-specificity have the potential to be superior to ABA in tuning crops into more water-efficiency for securing yield stability in water-restricted environments.

## Data availability statement

The original contributions presented in the study are included in the article/**Supplementary Material**. Further inquiries can be directed to the corresponding author.

## Author contributions

ZY, EG, and JF conceived and designed different parts of the research. AC, HH, SH, and SL supervised the experiments. JR performed yeast assays with contributions of MR. ID performed phosphatase assays. JL carried out protoplast assays and performed physiological analyses together with ZY. JR, ID, JL, and ZY analyzed the data. ZY wrote the article with contributions of all the authors. All authors contributed to the article and approved the submitted version.
