# Supplementary material for: Abscisic acid agonists suitable for optimizing plant water use
Source: Front Plant Sci. 2023 Jan 19;13:1071710. doi: 10.3389/fpls.2022.1071710 (PMC9894685; doi:10.3389/fpls.2022.1071710)
Supplement: Supplementary file 1 [file DataSheet_1.docx]

Supplementary figures and legends

|  |
| --- |
| **Supplementary Fig. S1** De-esterification of CCPs by nonspecific esterase. CCPs (A to C, G to I, and M to O) and CCPs treated with pig liver esterase (D to F, J to L, and P to R) were separated by HPLC and detected by their absorbance at 260 nm. De-esterified CCPs are indicated as “de-CCPs” above their corresponding peaks. The conversion rate of CCPs is calculated as the ratio of peak area of de-CCPs to the total peak area of de-CCPs and residual CCPs. CCP3 is the methyl-esterified form of CCP2. While CCP3 was completely converted into CCP2 by the esterase, CCP4 to CCP9 were only partially de-esterified. |

|  |
| --- |
| **Supplementary Fig. S2** The *in vitro* regulation of ABA receptor complex by cyano cyclopropyl compounds. Inhibition of ABI2 phosphatase by ABA (filled circles), CCP1 to CCP9 (open symbols as indicated) in the presence of RCAR1 (A,D,G), RCAR8 (B,E,H), and RCAR11 (C,F,I). The *in vitro* analysis was performed with 100 nM ABI2 and 200 nM RCAR at different ligand concentrations. (A to I) n = 3 replicates, mean ± SD. |

| 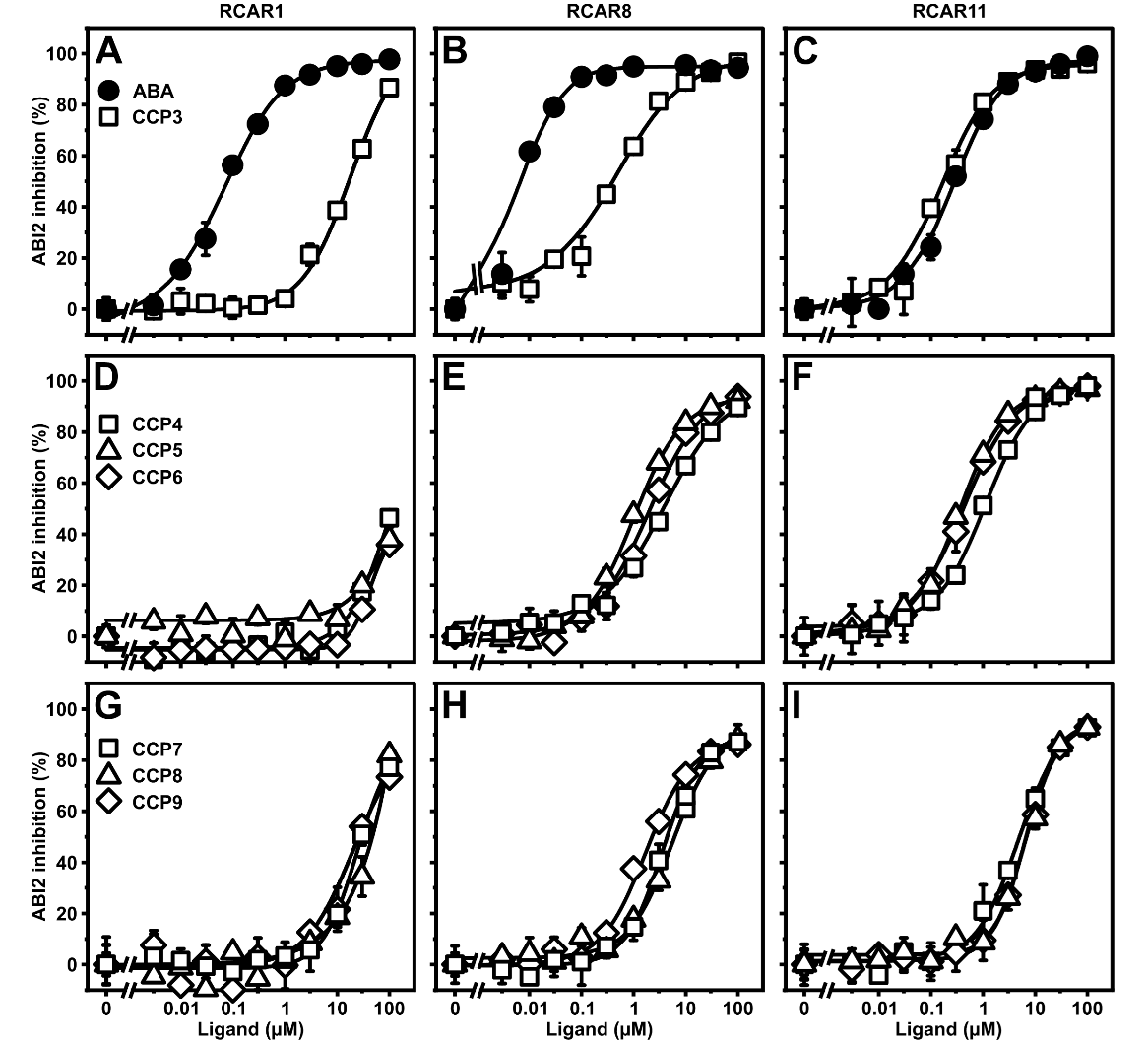 |
| --- |
| **Supplementary Fig. S3** The *in vitro* analysis of ABA receptor complex regulation by CCP compounds after esterase treatment. Inhibition of ABI2 phosphatase by ABA (filled circles), CCP3 to CCP9 (open symbols as indicated) in the presence of RCAR1 (A,D,G), RCAR8 (B,E,H), and RCAR11 (C,F,I). The *in vitro* analysis was performed as described in Supplementary Fig. S2. The CCPs were treated with pig liver esterase prior to the assays. The ligand concentration refers to the combined level of de-esterified and esterified CCP. (A to I) n = 3 replicates, mean ± SD. |

|  |
| --- |
| **Supplementary Fig. S4** Induction of ABA signaling in Arabidopsis cells by de-esterified CCP3. Regulation of ABA responsive expression of the LUC reporter was analyzed in Arabidopsis *aba2-1* mesophyll protoplasts by ectopic expression of 3 µg *RCAR11* and 0.1 µg *ABI2* (90% inhibition of basal ABA signaling) and in the absence and presence of 10 µM ABA, CCP3, and de-esterified CCP3 as indicated. Empty vector (E.V.) in the absence of ligand served as control and was set to 1 (5.7 × 10^3^ relative light units per relative fluorescence). “+” and “-” refer to the presence and absence of effector DNA, PLE, or ligands. Each data point represents the mean ± SD of three independent transformations plus three technical replicates per data point. |

|  |
| --- |
| **Supplementary Fig. S5** CCP1 and CCP5 have higher and longer-lasting efficacy than ABA in the control of Arabidopsis transpiration. (A,B) Time course of leaf temperature increase of plants treated with A) 10 µM or B) 30 µM solution of ABA (filled circles), CCP1 (open circles), or CCP5 (open squares). Thermal images were taken before the single treatment of 35-day-old plants over 19 consecutive days. The values of leaf temperature were determined based on the thermal images and were expressed as the temperature difference compared to the mock-treated plants (at days indicated in A and B with values of 22.2 ± 0.02 °C; 21.4 ± 0.04 °C; 22.1 ± 0.05 °C; 21.9 ± 0.02 °C; 22.0 ± 0.09; 21.7 ± 0.05; 21.7 ± 0.05; 21.6 ± 0.06; 20.8 ± 0.19 °C, respectively). Plants with different treatments were placed on trays at randomized positions. The growth conditions were described as in Fig. 5. (A,B) n = 5 biological replicates, mean ± SEM. |
