# Supplementary material for: Abscisic acid agonists suitable for optimizing plant water use
Source: Front Plant Sci. 2023 Jan 19;13:1071710. doi: 10.3389/fpls.2022.1071710 (PMC9894685; doi:10.3389/fpls.2022.1071710)
Supplement: Supplementary file 2 [file DataSheet_2.docx]

**Supplementary Tables**

Supplementary Table 1 Statistical significance of RCAR-ABI2-mediated reporter expression in Fig. 2A to 2C.

|  | **RCAR11-ABI2** | | | | | | **RCAR1-ABI2** | | | | | | **RCAR8-ABI2** | | | | | |
| --- | --- | --- | --- | --- | --- | --- | --- | --- | --- | --- | --- | --- | --- | --- | --- | --- | --- | --- |
| **Ligands (µM)** | 0.1 | 0.3 | 1 | 3 | 10 | 30 | 0.1 | 0.3 | 1 | 3 | 10 | 30 | 0.1 | 0.3 | 1 | 3 | 10 | 30 |
| **CCP1 to ABA** | ns | ns | ns | s | ns | s | ns | ns | ns | s | ns | s | ns | ns | ns | s | ns | s |
| **CCP2 to ABA** | ns | ns | s | s | s | s | ns | ns | s | s | s | s | ns | ns | s | s | s | s |
| **CCP2 to CCP1** | ns | ns | ns | ns | s | ns | ns | ns | ns | ns | s | ns | ns | ns | ns | ns | s | ns |

P < 0.05 (one-way ANOVA) is considered as a significant difference and expressed as “s”. Otherwise, the difference is regarded as insignificant indicated as “ns”.

Supplementary Table 2 Statistical significance of RCAR-HAB1-mediated reporter expression in Fig. 2D to 2E.

|  | **RCAR11-HAB1** | | | | | | **RCAR1- HAB1** | | | | | | **RCAR8- HAB1** | | | | | |
| --- | --- | --- | --- | --- | --- | --- | --- | --- | --- | --- | --- | --- | --- | --- | --- | --- | --- | --- |
| **Ligands (µM)** | 0.1 | 0.3 | 1 | 3 | 10 | 30 | 0.1 | 0.3 | 1 | 3 | 10 | 30 | 0.1 | 0.3 | 1 | 3 | 10 | 30 |
| **CCP1 to ABA** | ns | ns | ns | s | ns | s | ns | ns | ns | s | s | s | s | s | s | s | s | ns |
| **CCP2 to ABA** | ns | ns | ns | ns | ns | s | ns | ns | ns | s | s | s | s | s | s | s | s | s |
| **CCP2 to CCP1** | ns | ns | ns | ns | ns | ns | ns | ns | ns | ns | ns | s | ns | ns | ns | s | s | s |

P < 0.05 (one-way ANOVA) is considered as a significant difference and expressed as “s”. Otherwise, the difference is regarded as insignificant indicated as “ns”.

Supplementary Table 3 Statistical significance of RCAR11-mediated inhibition of ABI2 phosphatase activity in Fig. 3A.

| **Ligands (µM)** | **0.003** | **0.01** | **0.03** | **0.1** | **0.3** | **1** | **3** | **10** | **30** | **100** |
| --- | --- | --- | --- | --- | --- | --- | --- | --- | --- | --- |
| **CCP1 to ABA** | ns | ns | ns | ns | ns | ns | ns | ns | ns | ns |
| **CCP2 to ABA** | ns | ns | ns | ns | s | ns | ns | ns | ns | ns |
| **CCP2 to CCP1** | ns | ns | ns | s | s | ns | ns | ns | ns | ns |

P < 0.05 (one-way ANOVA) is considered as a significant difference and expressed as “s”. Otherwise, the difference is regarded as insignificant indicated as “ns”.

Supplementary Table 4 Statistical significance of RCAR1-mediated inhibition of ABI2 phosphatase activity in Fig. 3B.

| **Ligands (µM)** | **0.003** | **0.01** | **0.03** | **0.1** | **0.3** | **1** | **3** | **10** | **30** | **100** |
| --- | --- | --- | --- | --- | --- | --- | --- | --- | --- | --- |
| **CCP1 to ABA** | ns | s | s | s | s | s | s | s | s | s |
| **CCP2 to ABA** | ns | ns | s | s | s | s | s | s | s | s |
| **CCP2 to CCP1** | ns | ns | ns | s | s | ns | ns | ns | ns | ns |

P < 0.05 (one-way ANOVA) is considered as a significant difference and expressed as “s”. Otherwise, the difference is regarded as insignificant indicated as “ns”.

Supplementary Table 5 Statistical significance of RCAR8-mediated inhibition of ABI2 phosphatase activity in Fig. 3C.

| **Ligands (µM)** | **0.003** | **0.01** | **0.03** | **0.1** | **0.3** | **1** | **3** | **10** | **30** | **100** |
| --- | --- | --- | --- | --- | --- | --- | --- | --- | --- | --- |
| **CCP1 to ABA** | ns | s | s | s | s | s | ns | ns | ns | ns |
| **CCP2 to ABA** | s | s | s | s | s | s | s | ns | ns | ns |
| **CCP2 to CCP1** | ns | ns | s | s | s | s | s | ns | ns | ns |

P < 0.05 (one-way ANOVA) is considered as a significant difference and expressed as “s”. Otherwise, the difference is regarded as insignificant indicated as “ns”.

Supplementary Table 6 Statistical significance of RCAR11-mediated inhibition of HAB1 phosphatase activity in Fig. 3D.

| **Ligands (µM)** | **0.003** | **0.01** | **0.03** | **0.1** | **0.3** | **1** | **3** | **10** | **30** | **100** |
| --- | --- | --- | --- | --- | --- | --- | --- | --- | --- | --- |
| **CCP1 to ABA** | ns | ns | ns | ns | ns | ns | ns | ns | ns | ns |
| **CCP2 to ABA** | ns | ns | ns | ns | s | ns | ns | ns | ns | ns |
| **CCP2 to CCP1** | ns | ns | ns | s | s | ns | ns | ns | ns | ns |

P < 0.05 (one-way ANOVA) is considered as a significant difference and expressed as “s”. Otherwise, the difference is regarded as insignificant indicated as “ns”.

Supplementary Table 7 Statistical significance of RCAR1-mediated inhibition of HAB1 phosphatase activity in Fig. 3E.

| **Ligands (µM)** | **0.003** | **0.01** | **0.03** | **0.1** | **0.3** | **1** | **3** | **10** | **30** | **100** |
| --- | --- | --- | --- | --- | --- | --- | --- | --- | --- | --- |
| **CCP1 to ABA** | ns | s | s | s | s | s | ns | ns | ns | ns |
| **CCP2 to ABA** | s | s | s | s | s | s | ns | ns | ns | ns |
| **CCP2 to CCP1** | ns | ns | ns | ns | ns | ns | s | s | s | s |

P < 0.05 (one-way ANOVA) is considered as a significant difference and expressed as “s”. Otherwise, the difference is regarded as insignificant indicated as “ns”.

Supplementary Table 8 Statistical significance of RCAR8-mediated inhibition of HAB1 phosphatase activity in Fig. 3F.

| **Ligands (µM)** | **0.003** | **0.01** | **0.03** | **0.1** | **0.3** | **1** | **3** | **10** | **30** | **100** |
| --- | --- | --- | --- | --- | --- | --- | --- | --- | --- | --- |
| **CCP1 to ABA** | s | s | s | s | s | ns | ns | ns | ns | ns |
| **CCP2 to ABA** | s | s | s | s | s | s | s | ns | ns | ns |
| **CCP2 to CCP1** | ns | ns | s | s | s | s | ns | ns | ns | ns |

P < 0.05 (one-way ANOVA) is considered as a significant difference and expressed as “s”. Otherwise, the difference is regarded as insignificant indicated as “ns”.

Supplementary Table 9 Statistical significance of leaf growth of ligand-treated plants compared to mock-treated plants in Fig. 6A.

|  | | **Days under the progressive drought** | | | | | | | |
| --- | --- | --- | --- | --- | --- | --- | --- | --- | --- |
| **Ligands** | **Concentration** | **0** | **6** | **12** | **18** | **24** | **30** | **36** | **42** |
| **ABA** | **10 µM** | ns | ns | ns | ns | ns | ns | ns | ns |
| **ABA** | **30 µM** | ns | ns | ns | ns | s | s | s | ns |
| **CCP2** | **10 µM** | ns | ns | ns | ns | ns | ns | s | s |
| **CCP2** | **30 µM** | ns | ns | ns | ns | ns | ns | s | s |
| **CCP3** | **10 µM** | ns | ns | ns | ns | ns | s | s | s |
| **CCP3** | **30 µM** | ns | ns | ns | ns | ns | s | s | s |

P < 0.05 (one-way ANOVA) is considered as a significant difference and expressed as “s”. Otherwise, the difference is regarded as insignificant indicated as “ns”.
